# *EFF*ects of *E*xposure and *C*ognitive behavioral *T*herapy for chronic *BACK* pain (“EFFECT-BACK”): study protocol for a randomized controlled trial

**DOI:** 10.1186/s13063-024-08017-9

**Published:** 2024-03-11

**Authors:** Rabea Vogt, Julia Haas, Lukas Baumann, Anja Sander, Christina Klose, Jenny Riecke, Winfried Rief, Ulrike Bingel, Dustin Maser, Michael Witthöft, Jens Keßler, Marco Richard Zugaj, Beate Ditzen, Julia Anna Glombiewski

**Affiliations:** 1 Department of Psychology, University of Kaiserslautern-Landau (RPTU), Kaiserslautern, Germany; 2 Institute of Medical Biometry (IMBI), University of Heidelberg, Heidelberg, Germany; 3 Department of Clinical Psychology and Psychotherapy, Philipps - University of Marburg, Marburg, Germany; 4 Department of Neurology, Center for Translational Neuro- and Behavioural Sciences, University Hospital Essen, University Duisburg Essen, Essen, Germany; 5 Department of Clinical Psychology, Psychotherapy, and Experimental Psychopathology, Johannes Gutenberg-University Mainz, Mainz, Germany; 6 Heidelberg University, Medical Faculty Heidelberg, Department of Anesthesiology, Heidelberg, Germany; 7 Institute of Medical Psychology, Center for Psychosocial Medicine, Heidelberg University Hospital, Heidelberg, Germany

**Keywords:** Chronic back pain, Cognitive behavioral therapy, Exposure therapy, Fear avoidance, Randomized controlled trial, Clinical trial, Study protocol

## Abstract

**Introduction:**

Chronic back pain is a widespread medical condition associated with high socioeconomic costs and increasing prevalence. Despite the advanced implementation of multidisciplinary approaches, providing a satisfactory treatment offer for those affected is often not possible. Exposure therapy (EXP) promises to be an effective and economical form of treatment and in a previous pilot study showed to be superior to cognitive behavioral therapy (CBT) in reducing perceived limitations of movement. The current study aims to further compare the efficacy of both treatment methods and identify those patient groups that particularly benefit from EXP.

**Methods:**

The general objective of this randomized multicenter clinical trial (targeted *N* = 380) is to improve and expand the range of treatments available to patients with chronic back pain. As the primary objective of the study, two different psychological treatments (EXP and CBT) will be compared. The primary outcome measure is a clinically significant improvement in pain-related impairment, measured by the QPBDS, from baseline to 6-month follow-up. Secondary outcome measures are absolute changes and clinically significant improvements in variables coping, psychological flexibility, depressiveness, catastrophizing, exercise avoidance and fear of exercise, and intensity of pain. Participants are recruited in five psychological and medical centers in Germany and receive ten sessions of manualized therapy by trained licensed CBT therapists or clinical psychologists, who are currently in their post-gradual CBT training. Potential predictors of each treatment’s efficacy will be explored with a focus on avoidance and coping behavior.

**Conclusion:**

This study will be the first RCT to compare CBT and EXP in chronic back pain in a large sample, including patients from different care structures due to psychological and medical recruitment centers. By identifying and exploring potential predictors of symptom improvement in each treatment group, this study will contribute to enable a more individualized assignment to treatment modalities and thus improves the care situation for chronic back pain and helps to create a customized treatment program for subgroups of pain patients. If our findings confirm EXP to be an efficacious and efficient treatment concept, it should gain more attention and be further disseminated.

**Trial registration:**

ClinicalTrials.gov NCT05294081. Registered on 02 March 2022.

## Administrative information

Note: the numbers in curly brackets in this protocol refer to SPIRIT checklist item numbers. The order of the items has been modified to group similar items (see http://www.equator-network.org/reporting-guidelines/spirit-2013-statement-defining-standard-protocol-items-for-clinical-trials/).

**Table:** 

Title {1}	*EFF*ects of *E*xposure and *C*ognitive behavioral *T*herapy for chronic *BACK* pain (“EFFECT-BACK”): study protocol for a randomized-controlled trial
Trial registration {2a and 2b}.	NCT05294081 [ClinicalTrials.gov] [registered before the start of inclusion; 02-03-2022].
Protocol version {3}	Version 2.0 22^nd^ July 2022
Funding {4}	German Research Foundation (DFG, GL 607/8-1)
Author details {5a}	Rabea Vogt, Department of Psychology, University of Kaiserslautern-Landau (RPTU), Germany; Julia Haas, Department of Psychology, University of Kaiserslautern-Landau (RPTU), Germany; Lukas Baumann, Institute of Medical Biometry (IMBI), University of Heidelberg, Heidelberg, Germany; Anja Sander, Institute of Medical Biometry (IMBI), University of Heidelberg, Heidelberg, Germany; Christina Klose, Institute of Medical Biometry (IMBI), University of Heidelberg, Heidelberg, Germany; Jenny Riecke, Department of Clinical Psychology and Psychotherapy, Philipps - University of Marburg, Marburg, Germany; Winfried Rief, Department of Clinical Psychology and Psychotherapy, Philipps- University of Marburg, Marburg, Germany; Ulrike Bingel, Department of Neurology, Center for Translational Neuro- and Behavioural Sciences, University Hospital Essen, University Duisburg Essen, Germany; Dustin Maser, Department of Neurology, Center for Translational Neuro- and Behavioural Sciences, University Hospital Essen, University Duisburg Essen, Germany; Michael Witthöft, Department of Clinical Psychology, Psychotherapy, and Experimental Psychopathology, Johannes Gutenberg-University Mainz, Mainz, Germany; Jens Keßler, Heidelberg University, Medical Faculty Heidelberg, Department of Anesthesiology, Heidelberg, Germany; Marco Richard Zugaj, Heidelberg University, Medical Faculty Heidelberg, Department of Anesthesiology, Heidelberg, Germany; Beate Ditzen, Institute of Medical Psychology, Center for Psychosocial Medicine, Heidelberg University Hospital, Heidelberg University, Heidelberg, Germany; Julia Anna Glombiewski, Department of Psychology, University of Kaiserslautern-Landau (RPTU), Germany.
Name and contact information for the trial sponsor {5b}	Trial Sponsor: RPTU Kaiserslautern – Landau (former University of Koblenz – Landau) Fortstraße 7 76829 Landau, Germany
Role of sponsor {5c}	The funders did not play a role in the design of the study, data collection, analysis, and interpretation of the data, or in the writing of the manuscript. The sponsor RPTU Kaiserslautern – Landau (former University of Koblenz – Landau) is responsible for the design of the study; the collection, analysis, and interpretation of the data; and the writing of the manuscript.

## Introduction

### Background and rationale {6a}

Chronic back pain (CBP) challenges health systems and care structures worldwide [1–3]. Despite medical advances, CBP prevalence rates continue to increase [4, 5] and are one of the leading causes of medical costs, disability, and absenteeism [3, 6]. In 2020, 15.5% of the German population suffered from CBP [7] and 19% of adult Europeans [2]. However, the most common treatments include injections or surgery, which often show no substantial improvements and limited efficacy [8]. Pharmacotherapy shows small beneficial effects but also brings along a number of adverse events [9, 10]. It is therefore crucial to establish additional, more effective treatment options.

#### Cognitive behavioral therapy (CBT)

Cognitive behavioral therapy (CBT) and multimodal approaches including psychological components have been demonstrated to improve CBP and associated long-term impairment [11, 12]. The NICE guidelines for primary CBP approve that there is evidence of CBT efficacy in CBP treatment but still recommend further research [13], especially, given that previous studies have low power due to small sample sizes and do not provide the opportunity to perform subgroup analyses. Furthermore, the effects of psychological approaches are rather moderate and do not justify the high costs of inpatient care to enable multidisciplinary treatments [14, 15]. This emphasizes the need for more novel approaches to enable individualized, multimodal treatment programs. The integration of primary care and behavioral health therapies could lead to better treatment outcomes [9].

#### Graded exposure in vivo (EXP)

Graded exposure in vivo (EXP) is a more recent approach in psychological treatment for chronic pain, which specifically addresses the avoidance of physical activity, but is so far rarely used in clinical practice. Three randomized controlled trials (RCTs) on EXP in CBP show positive effects on pain-related impairment, kinesiophobia, depressive mood, pain catastrophizing, and perceived harmfulness of activity [16–18]. While EXP was not superior to graded activity in terms of pain-related impairment [17, 18], it is important to note the rather small sample sizes and short follow-up periods in these studies. A recent pilot study of our team adds to the previous RCTs and supports their promising results.

#### Pilot study

In the pilot study [19], researchers investigated the feasibility of EXP in a psychological outpatient setting among 88 individuals with high fear avoidance levels and explored the optimal treatment duration of EXP. A short exposure-based treatment program with 10 sessions (EXP-short) and a longer exposure-based treatment program with 15 sessions (EXP-long) were carried out and compared to a 15-session CBT standard program. This trial demonstrated that EXP could be administered safely in the outpatient psychological setting. While CBT was more effective than EXP in improving coping strategies, EXP was more effective than CBT in reducing movement-related impairment. Furthermore, EXP-short outperformed EXP-long in efficiency after 10 sessions, meaning that individuals improved faster when offered fewer sessions. There were significantly more dropouts under the EXP condition, which is consistent with the results of the RCTs described above. EXP appears to be effective and efficient, but also more challenging for patients.

Further analyses of the pilot study data show in an 8-year follow-up that both CBT and EXP provide sustained positive effects for patients with chronic back pain [20]. Furthermore, analyses of these data show that a behavioral measure, the “BAT-BACK” test [21], was successful in identifying participants who benefited from EXP in terms of reducing pain-related impairment [22]. This finding suggests that participants who show greater avoidance of physical activities in the BAT-BACK benefit the most from EXP treatment. Therefore, in the future, EXP therapy could be a customized treatment option to achieve quick and specific symptom improvement in subgroups of patients with CBP. Consequently, larger RCTs are needed to further clarify the beneficial effects of EXP.

#### Need for trial

CBP is a widespread disease that leads to high socioeconomic costs with only a few available potentially effective treatment options. Psychological treatments, including CBT, are recommended; however, studies with enough statistical power are lacking. EXP has also demonstrated efficacy as a treatment option for CBP in previous RCTs, but the exact advantages over CBT and the subgroups for which it works best need to be investigated in more detail. This implies that studies with higher power, comparative treatment options, and samples reflecting the reality of care are needed.

For this reason, the current multicenter RCT aims to replicate and extend the results of our pilot study [19]. This study aims to compare an already established psychological treatment form (CBT) with a novel and innovative treatment form (EXP) in a large CBP sample. Furthermore, the goal of this research is to explore which patient subgroups benefit from which treatment conditions. By including both psychological and medical treatment centers, the sample will reflect the clinical reality and population characteristics of different care structures.

### Objectives {7}

The primary objective of the project is to improve and expand the range of treatments for patients with CBP. The study aims to compare the effects of two different psychological methods (CBT and EXP) in the treatment of CBP on the participants’ perceived pain-related impairment. A secondary objective is to explore potential predictors of treatment effects by analyzing the role of movement avoidance and coping.

As a result, the following is the hypothesis for this study:

H_1_: EXP is more successful than CBT in reducing pain-related impairments.

Exploratory with regard to “tailored treatment,” we expect that

- Individuals who have higher scores on the behavioral avoidance test BAT-BACK will rather benefit from EXP than from CBT
- Individuals who have lower scores on coping will rather benefit from CBT than EXP in terms of reduction of the primary outcome measure (disability)

Exploratively, we also look at absolute changes and clinically significant improvements in variables coping, psychological flexibility, depressiveness, catastrophizing, exercise avoidance and fear of exercise, and intensity of pain.

### Trial design {8}

**Fig. 1:**
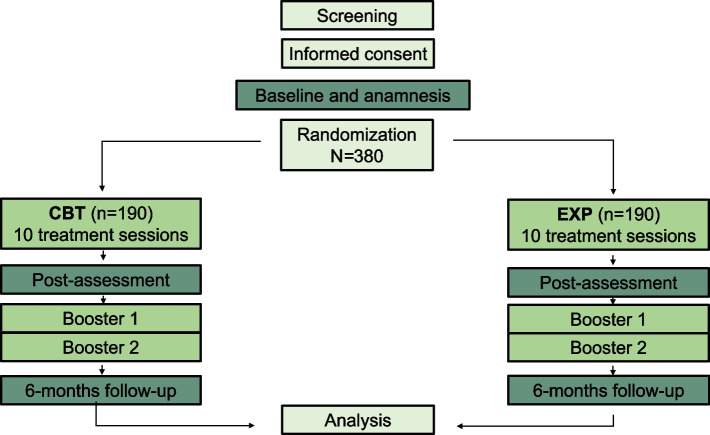
Treatment arms and sample sizes. CBT: cognitive behavioral therapy; EXP: graduated exposure in vivo

This is a prospective multicenter, randomized controlled, open-label, two-arm intervention study with a parallel group design that will be conducted in five study centers in Germany. A total of 380 patients with CBP will be included, 190 patients per study arm. Participants will be randomly allocated to the two conditions. The intervention group will receive ten sessions of EXP therapy and the active control group will receive ten sessions of CBT. Primary and secondary outcomes will be measured before randomization (baseline), after completion of the ten treatment sessions (post-assessment), and at 6-month follow-up. In each group, two booster sessions will be implemented between post-assessment and follow-up (Fig. 1).

## Methods: participants, interventions, and outcomes

### Study setting {9}

Five German academic health facilities will serve as study centers: the outpatient clinic for psychotherapy of the RPTU Kaiserslautern – Landau (former Koblenz – Landau), the outpatient clinic for psychotherapy of the Johannes Gutenberg University Mainz, the outpatient clinic for psychotherapy of the Philipps University Marburg, the multidisciplinary pain center of the University Essen, and the multidisciplinary pain center of the University Heidelberg. This means that both psychological and medical centers are involved in patient recruitment and the implementation of study therapies. In all centers, treatment will be offered in an outpatient setting.

### Eligibility criteria {10}

Adults (≥ 18 years) with chronic back pain, defined as back pain on most days of the week for a duration of at least 6 months, and a sufficient level of impairment, defined by the Quebec Pain Disability Scale (QBPDS [23], score ≥ 15), will be included. Written informed consent will be obtained from all participants before they are included.

Patients who meet at least one of the following aspects will be excluded from participation (exclusion criteria): back surgery during the last 6 months or planned back surgery, medical contraindications as suggested by previously defined red flags and medical consultation, insufficient German reading and/or speaking, current pregnancy, severe alcohol and/or drug addiction, psychotic symptoms, parallel psychological treatment, physical inability to attend sessions, or parallel participation in another intervention study. Red flags were determined in cooperation with a physician specialized on CBP as fractures (severe or minor), evidence of tumors or infections, history of tumors, condition after bacterial infection, increased pain when lying on the back, severe pain at night, or evidence of nerve compression/caudal syndrome (neurological deficits, bladder/bowel weakness).

The study therapists will be licensed CBT therapists or clinical psychologists, who are currently in their post-gradual CBT training. Before treating study patients, all of them will undergo a study-specific training by the principal investigator (JAG) to educate them about chronic pain, the two treatment conditions, and the study procedures.

### Who will take informed consent? {26a}

All interested patients who meet the eligibility criteria and do not meet the exclusion criteria are invited to a detailed information session with a study staff member to inform them about the study verbally and in writing. They will be informed about the following: (a) the research question and its scientific relevance, (b) the procedure and duration of participation, (c) potential risks or side effects, (d) expected benefits, (e) collection and processing of personal data, and (f) their right to withdraw from participation at any time without giving reasons and without implied negative consequences. There will be sufficient time and opportunity to clarify any questions or concerns. If the patient is willing to participate in the trial, written informed consent will be obtained.

### Additional consent provisions for collection and use of participant data and biological specimens {26b}

Not applicable, the trial does not involve collecting biological specimens.

## Interventions

### Explanation for the choice of comparators {6b}

The experimental intervention EXP will be compared to the active intervention control group CBT which is a typical treatment component in multidisciplinary inpatient settings of chronic pain treatment. CBT as a “broad spectrum” treatment aims to teach coping strategies and was found to be beneficial for most patients with CBP with only a few side effects and low dropout rates [19]. In the pilot study, CBT improved different outcome variables than EXP, and thus, researchers are interested in investigating these differences further to determine if they will persist in the larger trial. Due to ethical reasons, researchers decided not to include a no-treatment control group.

### Intervention description {11a}

#### Cognitive behavioral therapy and graduated exposure in vivo

Both CBT and EXP aim to provide patients with strategies for better pain management and to improve their well-being and quality of life. Treatment strategies differ in their focus and method: CBT supports patients in developing an adaptive coping style to deal with pain. It focuses on problem-solving skills, activity pacing, relaxation, and attention management. EXP for chronic pain aims to reduce pain-related impairment by managing fear of movement and increasing confidence in one’s own physical resilience.

Manuals for both treatment arms had already been designed for the previous pilot study [19]. Based on the experience during the pilot study, they were afterwards revised and refined through several meetings and continuous exchange between JAG and JR who were the principal investigator and the study coordinator of the pilot study and who have a lot of clinical experience in the treatment of CBP. Both treatment manuals provide guidelines for ten treatment sessions and two booster sessions. For ethical reasons and to reflect the reality of care, they also involve the option of up to two so-called joker sessions to allow addressing therapeutically urgent topics beyond the manual-specific content, like management of acute crises. A detailed overview of the content of each treatment session is given in Table 1.

**Table 1:** Overview of treatment sessions

Session	CBT	EXP
1	Goal setting; working alliance	Goal setting; working alliance
2	Pain education I	Pain education I
3	Pain education II: psychophysiological model of somatoform disorder	Pain education II: fear avoidance model
4	Relaxation	Exposure 1
5	Graded activity I	Exposure 2
6	Graded activity II	Exposure 3
7	Attention shifting	Exposure 4
8	Cognitive restructuring	Exposure 5
9	Cognitive restructuring	Exposure 6
10	Reflection and transition to the self-management phase	Reflection and transition to the self-management phase
Booster 1	Support and reflection	Support and reflection
Booster 2	Support and reflection	Support and reflection
Joker 1	In case of need and unexpected therapeutic issues outside of the manual	In case of need and unexpected therapeutic issues outside of the manual
Joker 2	In case of need and unexpected therapeutic issues outside of the manual	In case of need and unexpected therapeutic issues outside of the manual

*CBT* Cognitive behavioral therapy, *EXP* Graduated exposition in vivo

### Criteria for discontinuing or modifying allocated interventions {11b}

No criteria have been defined and there is no provision for changing treatment arms. Should reasons occur during treatment that make a strictly manualized treatment appear ethically untenable (e.g., suicidality), the therapist and supervisor (see the following section) may decide together on a change to standard outpatient treatment.

### Strategies to improve adherence to interventions {11c}

To verify and ensure adherence to the treatment manuals, all therapists will receive study-specific training. Therapy sessions will be videotaped and evaluated, and therapists will rate their adherence for each treated patient on a regular basis. The evaluation of adherence to treatment is based on the method of assessing treatment delivery in clinical trials [24]. Accordingly, manual adherence is defined as the presence of at least 70% of the essential treatment elements. Treatment contamination is defined as the presence of at least 10% of the prohibited treatment elements. Treatment differentiation is considered achieved when more than 90% of the sessions have been correctly classified as EXP or CBT. According to Leeuw et al. [24], each treatment element is assigned to one of the following categories for each treatment condition: (a) essential and specific, (b) essential but not specific, (c) compatible but not essential and not unique, and (d) prohibited. Additionally, all treatments are regularly supervised (every fourth session) by licensed supervisors experienced in CBT and EXP therapy to increase treatment quality and ensure fidelity to the manual.

### Relevant concomitant care permitted or prohibited during the trial {11d}

All adjuvant pain medications taken for at least 4 weeks prior to the baseline will be recorded and their continued intake will be allowed. If participants have just started a new medication regimen, the baseline measurements will be delayed by 4 weeks until it can be assumed that the medication regimen is stable, and the participant is habituated to the substance. Participants are asked not to change their medication regimen until follow-up. If a change is necessary, this will be documented and considered in the analysis. Participants are asked not to take medication on demand or emergency drugs as this could be a safety behavior that contradicts the basic principle of exposure. Other accompanying treatments, such as physical therapy or osteopathy, are allowed as this reflects the reality of clinical practice. Accompanying treatments are recorded and changes are monitored.

### Provisions for post-trial care {30}

No provisions are anticipated for post-trial care.

### Outcomes {12}

The *primary outcome* will be improvement in pain-related impairment between baseline and 6-month follow-up as assessed by the Quebec Back Pain Disability Scale (QBPDS [12]). Researchers will focus on clinically significant improvement in impairment using the two-step Jacobson and Truax (JT) method [25]. This method evaluates the reliability of symptom changes in the context of the sample’s overall symptom distribution. As compared to referring to statistical significance only, this approach is more conservative and allows researchers to draw conclusions that are clinically relevant and meaningful [15]. As in the pilot study, to define a reliable and clinically significant improvement in QBPDS, we will refer to the test-retest reliability of .92 and the pre-treatment mean of 45.6 (*SD* = 15.66) from a study about development and evaluation of the QBPDS and its psychometric properties [12]. Since no normative data exists for the QBPDS, Jacobson and Truax’s “criterion A” will be used, a criterion that relates exclusively to clinical distribution and was already used in the pilot study. “Criterion A” assesses whether a person deviates by more than two standard deviations from the mean of the patient group [21]. Based on the pilot study, the cut-off value for “criterion A” for QBPDS will be 14, i.e., an improvement of 14 or more will be considered clinically significant.

Absolute changes in the following variables from baseline to post-assessment and follow-ups serve as *secondary outcomes*: (a) pain-related disability measured by the QBPDS [12] and via the Pain Disability Index (PDI [26]); (b) coping strategies measured by the coping scale of the German Questionnaire for the Assessment of Pain Processing (Fragebogen zur Erfassung der Schmerzverarbeitung, FESV [27]); (c) depressed mood assessed by the depression subscale of the Anxiety and Depression Scale (HADS [16]); (d) catastrophizing thoughts measured by the Pain Catastrophizing Scale (PCS [28]); (e) fear avoidance measured by the Photo Series of Daily Activities (PHODA [29]); (f) fear of pain assessed by the Pain Anxiety Symptom Scale (PASS-20 [30]); (g) avoidance behavior measured by the Behavioral Avoidance Test—Back Pain (BAT-BACK [21]; (h) psychological flexibility assessed by the Psychological Inflexibility in Pain Scale (PIPS [31]); and (j) pain intensity and experienced impairment via an adapted 11-item scale from the German Pain Questionnaire (Deutscher Schmerzfragebogen, DSF [32]).

As with the primary outcome, the JT method [25] will be used. As in the pilot study, to define a reliable and clinically significant improvement in PDI [33], researchers will use the test-retest reliability of .91 from the study by Grönblad et al. [34] and the mean (*M* = 33.69, *SD* = 11.59) and normative population data (*M* = 6.8, *SD* = 11.4) from the study by Mewes et al. [35]. The reliable improvement criterion is set at 9.64 or more, and the threshold for clinically significant change is set at 10.51 (clinical distribution criterion only, “criterion A”). The reliable improvement criterion based on both clinical distribution and normative data (“criterion C”) is defined as an improvement of 20.13 or more. For the numerical pain intensity scale, an improvement of at least 1.5 points (or about 20%) is considered clinically significant [36].

Additionally, a safety assessment and recording of adverse side effects will follow every third session of therapy, using the Inventory for the Assessment of Negative Effects of Psychotherapy (INEP [37]). Furthermore, therapists complete a checklist after each therapy session to record adverse events and/or events that could influence the course of therapy. Demographic and anamnestic information including medical history will be assessed at baseline and follow-up by a questionnaire on the participants’ sociolegal situation, health care, and absences from work (module “S” of the DSF [32]).

### Participant timeline {13}

Those interested in the study will be screened for inclusion and exclusion criteria by phone or, in medical centers, directly on-site. Preliminary eligible patients will be invited for a personal and detailed information interview at the respective recruitment center. Potential study participants will receive detailed information on participation, final eligibility will be assessed, and open questions will be clarified. If all eligibility criteria are met and the patient agrees to participate by signing the informed consent, two appointments will follow for the baseline assessment. Afterwards, participants will be randomly assigned to one of the treatment arms and will receive 10 sessions of CBT or EXP therapy, respectively. During the course of each session, pain intensity (DSF scales [32]) and pain-related impairment (PDI [26]) will be measured. In every third session, the therapeutic relationship and potential side effects of psychotherapy (INEP [37]) will be assessed. In the EXP condition, a questionnaire on expectation violation (Rating of Expectancy Violation, REV) will be included in the sessions with exposure exercises. Concomitant treatments, pain medication, and unexpected events that could potentially affect our therapy will be monitored and recorded as they occur. After the treatment phase, participants will be invited to the post-assessment, and booster sessions with the therapists will take place at intervals of 1 and 3 months after the post-assessment. Follow-up is planned 6 months after post-assessment.

**Fig. 2:**
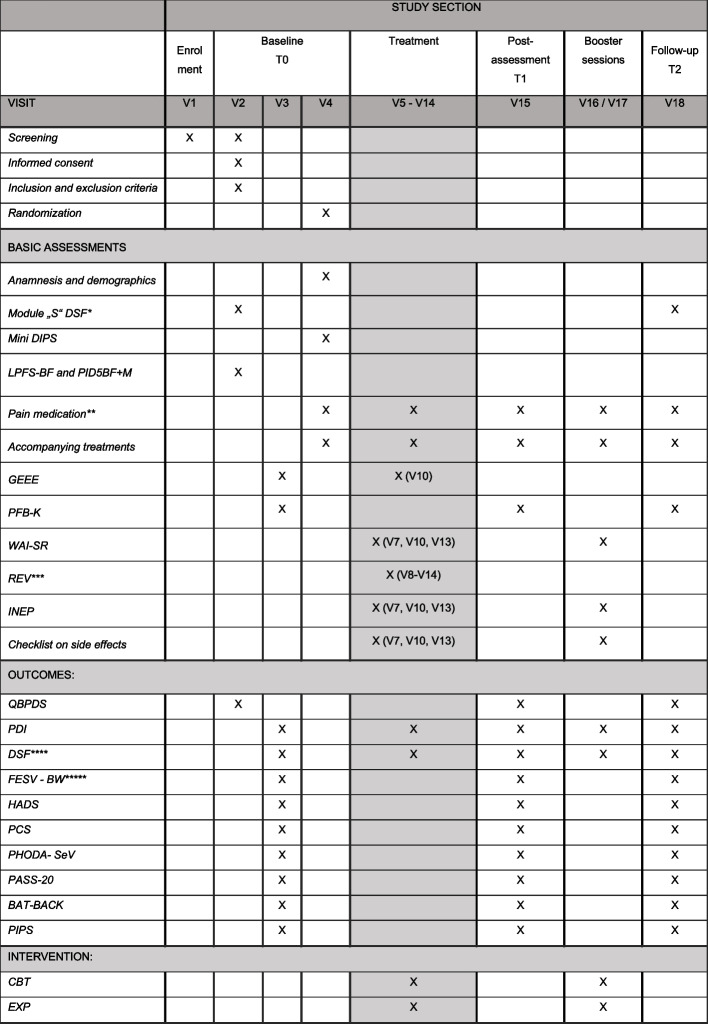
Visit plan. *CBT*, cognitive behavioral therapy; *EXP*, graduated exposure in vivo; *Module “S” DSF, module on sociolegal situation and absenteeism of the German Pain Questionnaire (Deutscher Schmerzfragebogen [32]); *Mini DIPS*, Diagnostische Kurzinterview bei psychischen Störungen; *LPFS-BF*, Level of Personality Functioning Scale - Brief Form [38]; *PID5BF+M*, Personality Inventory for DSM-5 - Brief Form Plus [39]; *GEEE*, generic rating scale for previous treatment experiences, treatment expectations, and treatment effects [40]; *PFB-K*, Partnerschaftfragebogen Kurzform [41] [31]; *WAI-SR*, Working Alliance Inventar short revised [42]; *REV*, Rating of Expectancy Violation; *INEP*, Inventar zur Erfassung Negativer Effekte von Psychotherapie [37]; QBPDS, Quebec Back Pain Disability Scale [23]; *PDI*, Pain Disability Index [26]; *FESV-BW*, Fragebogen zur Erfassung der Schmerzverarbeitung [13]; *HADS*, Hamilton Anxiety and Depression Scale [43]; *PCS*, Pain Catastrophizing Scale [28]; *PHODA-SeV*, Photograph Series of Daily Activities - Short Electronic Version [44]; *PASS-20*, Pain Anxiety Symptoms Scale [30]; *BAT-BACK*, Behavioral Avoidance Test—Back Pain [21]; *PIPS*, Psychological Inflexibility in Pain Scale **including adjuvant pain medication (antidepressants); ***only in EXP condition; ****adapted scales on pain intensity and experienced impairment of the German Pain Questionnaire (Deutscher Schmerzfragebogen [32]); ******coping scale

Figure 2 presents the participant timeline and the scheduling of the assessments and therapy sessions.

### Sample size {14}

Based on the results of the pilot study, 44% of the subjects in CBT and 65% of the subjects in the short EXP condition showed a clinically significant change in pain-related impairment [19], researchers suppose a conservative response rate of 60% in the EXP condition. Using a two-sided chi-square test to detect this effect at a significance level of 0.05 and a power of 80%, 152 patients per group are required. The use of a mixed logistic model that includes baseline QBPDS, HADS, BAT-BACK, and PHODA scores as fixed effects and the study center as a random effect in addition to the treatment group is expected to increase the power of the analysis. Based on comparable studies [16, 21] in this research area and the pilot study [19], a dropout rate of 20% is expected. To account for these expected dropouts, 380 participants (190 per group) will be randomized. The sample size calculations were performed with PASS 14.0.8 [45].

### Recruitment {15}

Participants will be recruited by approaching patients who receive medical care at our medical study centers or present for the first time. Psychology centers will recruit through self-initiated patient requests for psychotherapy in general and through self-initiated requests specifically for the study. Information flyers will be placed with local primary care physicians, physical therapists, orthopedists, and anesthesiologists. Newspaper articles and press releases will be prepared, and recruitment emails will be sent via university mailing lists. In addition, self-help CBP groups will be contacted and asked to disseminate study flyers in their networking sample.

## Assignment of interventions: allocation

### Sequence generation {16a}, concealment mechanism {16b}, and implementation {16c}

The allocation to the two treatment conditions is randomized. After the participants gave their informed consent and completed the baseline, they will be randomly assigned to the treatment conditions CBT or EXP in a 1:1 ratio by a study staff member using the centralized web-based tool Randomizer (www.randomizer.at). Randomization will be stratified by center and block wise to achieve equally sized groups per stratum. The block length will be determined by the biometrician of the study and kept confidential to prevent selection bias. Once a participant is allocated, their assigned therapist will be informed about their treatment condition.

## Assignment of interventions: blinding

### Who will be blinded {17a}

Due to the nature of the treatments, neither the patients nor the therapist can be blinded to the assigned treatment. Data analysts are blinded until database closure.

### Procedure for unblinding if needed {17b}

Not applicable as it is not a blinded study.

## Data collection and management

### Plans for assessment and collection of outcomes {18a}

Surveys will primarily be collected in digital form. If a participant prefers the paper-pencil version, it can be provided and then the data will be entered into the electronic database by the study staff.

#### Primary outcomes

The primary outcome, clinically significant pain-related disability, will be measured by the Quebec Pain Disability Scale (QBPDS [23]). This 20-item questionnaire shows strong psychometric properties with high internal consistency (*α* = 0.94) and good validity [46]. Items are scored from 0 to 5 (0 = not difficult at all, 5 = unable to do), and the item scores are summarized to a total score. Greater total scores reflect higher disability.

#### Secondary outcomes

Pain-related disability will also be assessed by the Pain Disability Index (PDI [26]). Each of the 7 items is scored from 0 to 10 (0 = no disability, 10 = maximum disability). Higher sum scores reflect greater interference of pain with daily activities and good validity and reliability have been demonstrated [34]. The average intensity of pain during the last 4 weeks will be assessed by an 11-point scale (0 = no pain, 10 = strongest pain) with three items of the German Pain Questionnaire (Deutscher Schmerzfragebogen, DSF [32]). A higher score reflects stronger pain; high reliability and validity of the DSF have been reported [32].

Change in coping strategies will be measured by the 24-item coping subscale of the German Questionnaire of Pain Processing (Fragebogen zur Erfassung der Schmerzverarbeitung, FESV-BW [27]). Each item is scored from 1 to 6 (1 = not true at all, 6 = completely true). Higher sum scores reflect a more frequent use of different coping strategies.

The change in emotional distress will be assessed by the depression subscale of the Hospital Anxiety and Depression Scale (HADS) [43]. Each item is scored from 0 to 3. Higher sum scores reflect greater anxiety or depression. Good reliability and validity have been demonstrated [47].

The change in pain catastrophizing will be measured by the Pain Catastrophizing Scale (PCS) which shows strong psychometric properties with high internal consistency (*α* = 0.92) and good validity [33]. Each item is scored on a scale of 0 to 4 (0 = not at all, 4 = all the time). Higher total scores reflect more catastrophizing thoughts.

Change in pain anxiety will be assessed by the Pain Anxiety Symptom Scale (PASS-20) [30]. Each item is scored from 0 to 5 (0 = never, 5 = always). Higher total scores reflect greater fear of pain. Good reliability (*α* = 0.90) and validity have been demonstrated [30].

The change in psychological inflexibility will be measured using the Psychological Inflexibility in Pain Scale (PIPS) [31]. Each item is scored from 1 to 7 (1 = never true, 7 = always true). Higher total scores reflect higher inflexibility regarding pain. The scale was reported to be a valid and reliable measure of avoidance [31].

Avoidance behavior will be assessed by the Behavioral Avoidance Test—Back Pain [21]. This behavioral test assesses pain-related avoidance behavior by direct observation. Participants are guided to imitate three movements with up to 10 repetitions: (1) bending forward, (2) lifting a crate (~8 kg), and (3) rotating. The degree of their avoidance and safety behavior is rated by the observer in three categories: (1) movement is performed as shown (score = 0), (2) movement is performed with safety behaviors (score = 1), and (3) avoiding the movement (score = 3). The range of possible total scores is 0 to 60. A score of 0 indicates that the participant did not avoid any of the movements or engage in safety behaviors. A score of 60 indicates that the participant avoided every movement.

Furthermore, the Photo Series of Daily Activities (PHODA-SeV) [44] will be used as an electronic tool to assess the perceived harmfulness of daily activities. Participants are instructed to sort photos showing daily activities on a 100-point visual analog scale from 0 (not harmful at all) to 100 (extremely harmful).

Strong psychometric properties, with high internal consistency (*α* = 0.92) and appropriate structural validity, have been demonstrated [44]. Higher scores reflect more perceived harm of daily activities.

Two additional questionnaires are amended by the sites, the generic rating scale for previous treatment experiences, treatment expectations, and treatment effects (GEEE, [40]) and a questionnaire assessing satisfaction in partnership (Partnerschaftsfragebogen – Kurzform, PFB-K, [41]).

### Plans to promote participant retention and complete follow-up {18b}

Participants will be informed about the study’s objective and relevance. The importance of completing the treatment and the follow-up evaluation, both for the study’s quality and for their personal chance to benefit from the therapy, will be emphasized.

### Data management {19}

The Institute of Medical Biometry (IMBI) in Heidelberg will be responsible for data management and for providing the required electronic infrastructure. The study data will be collected and managed using the REDCap (Research Electronic Data Capture [48]) system, a secure web-based application hosted by IMBI. All primary and secondary outcomes, as well as additional assessments and global instruments on study discontinuation, deviations from the protocol, concomitant treatments, and medication, will be entered into the participant’s electronic case report form (eCRF) by the responsible personnel or designated representative. The responsible staff or designated representative will be instructed to complete the eCRF sections as soon as possible after the information is collected, preferably on the same day that the participant appears for an examination, treatment, or measurement procedure. Any remaining entries must be completed immediately after follow-up and an explanation will have to be provided for any missing data. The completed eCRF must be reviewed and signed by the responsible member of the staff or the designated representative. The lead center will prepare detailed standardized operating procedures (SOPs) for each assessment and all study staff will be instructed in these procedures. All data will be pseudonymized and encrypted using Secure Socket Layer (SSL) technology, and the database server will be protected by a firewall. In REDCap, user roles and rights can be defined to ensure that only authorized users can enter and edit data. Access to participants’ data will be restricted to the respective center. Data changes will be logged along with a computerized time stamp on an audit trail. To ensure high data quality, rules for data validation will be defined in a data validation plan. The completeness and plausibility of the data will be ensured through edit checks during data entry and with the help of generated queries from validation programs. A tracking system for the eCRF data will be set up to ensure data is managed in a timely manner. Once all data are entered and no more changes to the database are needed, the eCRF data are locked and subsequently downloaded for the statistical analyses. All data management procedures will be carried out according to the written SOPs, which ensure efficient implementation and compliance with Good Clinical Practice [49]. At the end of the study, the data will be converted into different data formats for archiving and reuse.

### Confidentiality {27}

All people involved in the study are subject to confidentiality. All data will be collected and analyzed pseudonymously. Extensive measures will be taken to protect the data, especially personal data, against access by third parties. Local therapy files and study data will be stored in locked cabinets and will only be accessible to study staff and to the respective treating therapists who are subject to the legal duty of confidentiality. If participants withdraw their consent to the study, they will be asked about the reasons for their early dropout. The data collected so far will further be used and analyzed within the study, unless participants withdraw their consent to processing their data as well. If a participant only discontinues the study treatment, but not participation in general, further data required for the study can be collected and used.

All therapists, supervisors, and study assistants are also subject to medical confidentiality. No information collected during the therapy will be passed on to third parties unless the participants give their explicit written consent.

### Plans for collection, laboratory evaluation, and storage of biological specimens for genetic or molecular analysis in this trial/future use {33}

Not applicable as no biological specimens will be collected.

## Statistical methods

### Statistical methods for primary and secondary outcomes {20a}

The *primary outcome* (clinically relevant improvement in QBPDS scores) will be analyzed using a mixed logistic regression model. Treatment group and baseline scores of QPBDS, HADS, BAT-BACK, and PHODA will be considered as fixed effects. Additionally, center-specific random intercepts will be specified. The confirmatory test of the hypothesis will be carried out in the full analysis set by conducting a *z*-test to test whether the regression coefficient of the treatment group is different from 0. This test will be performed at a two-tailed significance level of .05.

As a supplementary analysis for the primary outcome, the primary logistic mixed regression model will be replicated, this time including a different depression covariate (depression as a binary variable, determined based on the clinical structured interview, instead of the baseline HADS score). In addition, another model will be calculated in which a change in medication as a binary variable will be included as a covariate. It should be noted that the covariate adjustment of logistic regression models will change the parameter to be estimated (“non-collapsibility”). In each case, the probability parameters refer to subgroups of participants who have the same expression of the variable to which conditionalization is applied [50]. Since the supplementary analyses will condition on other covariates, there will be a change in the true regression parameter for the treatment group.

Secondary endpoints will also be analyzed using mixed regression models with center-specific random intercepts that adjust for the respective baseline value and the HADS baseline value. Descriptive *p*-values and 95% confidence intervals for the treatment group difference will be calculated based on these models.

### Interim analyses {21b}

Not applicable since there will be no interim analyses regarding the main objective of the trial.

### Methods for additional analyses (e.g., subgroup analyses) {20b}

Exploratory absolute changes in pain-related disability, coping, depressed mood, catastrophizing, fear avoidance, fear of pain, avoidance behavior, and psychological flexibility as *secondary outcomes* will be analyzed using appropriate mixed regression models, adjusting for respective baseline scores. Center-specific random intercepts will also be specified in each model. Descriptive *p*-values and 95% confidence intervals will be calculated to determine the effect of the treatment group on any secondary outcomes**.**

To identify subgroups of participants who particularly benefit from EXP or from CBT and to test the related *explorative hypotheses* (individuals who show more avoidance behavior will be more likely to benefit from EXP than from CBT and individuals with lower coping skills are more likely to benefit from CBT than from EXP), a linear mixed regression model will be calculated in which the 6-month follow-up QBPDS score will be the dependent variable, and the treatment group, the baseline QBPDS score, the baseline HADS score, the baseline PHODA score, the baseline BAT-BACK score, and the baseline FESV score will serve as independent variables. In addition, interaction terms between the treatment group and BAT-BACK as well as the treatment group and FESV score will be included. Again, center-specific random intercepts will be specified. To analyze whether the BAT-BACK or FESV score is related to the treatment group, it will be tested whether the respective interaction terms are different from 0. The mixed regression model will also be used to estimate the effects of individualized treatment and to estimate optimal individualized treatment rules.

### Methods in analysis to handle protocol nonadherence and any statistical methods to handle missing data {20c}

The analysis will follow an intention-to-treat strategy. Missing values in the QPBDS (from which the primary outcome is derived) or in the HADS will be imputed for each item using the predictive mean matching (PMM) method. QPBDS (baseline and 6-month follow-up) and HADS, BAT-BACK, and PHODA (baseline only) will also be included in a multiple imputation model, as well as the study center as a random effect. For sensitivity analyses, a pattern mixture model for imputation will be used instead of the PMM model, and best-case and worst-case scenarios for imputation of missing values will be considered.

### Plans to give access to the full protocol, participant-level data, and statistical code {31c}

Non-identifiable data sets can be made available by the principal investigator upon reasonable request.

## Oversight and monitoring

### Composition of the coordinating center and trial steering committee {5d}

The coordination of this multicenter study will be carried out by the Working Group “Clinical Psychology and Psychotherapy” of the RPTU Kaiserslautern – Landau. The trial steering committee is composed of a principal investigator (PI) and assisting senior coordination staff from the lead study center. The PI will not only supervise all aspects of the study, but will also be responsible for the training and supervision of the study therapists. In each center, a study coordinator or study coordination team will be responsible for implementing the trial procedures on-site, recruiting participants, and organizing data entry. There will be a monthly online meeting of all centers for briefing, organizational issues, and clarification of questions. Data management will be performed by the IMBI of the University of Heidelberg. Clinical monitoring with on-site visits and auditions will be performed by the “Center for Methods, Diagnostics and Evaluations” of the RPTU Kaiserslautern – Landau in collaboration with IMBI’s central (statistical) monitoring.

### Composition of the data monitoring committee, its role, and reporting structure {21a}

The independent Data Safety and Monitoring Board (DSMB) will include a psychologist with practical and scientific experience in the field of chronic pain, a medical pain specialist, and a statistician. This board will be responsible for monitoring the progress of the study as defined by the milestones and, if necessary, reporting recommendations for adjustments to the implementation or termination of the study to the trial steering committee. The DSMB will also be responsible for reviewing the ethical conduct of the study and ensuring the participants’ safety. During the treatment period, there will be meetings with the study management every 6 months to inform the DSMB about the centers’ compliance with the study protocol, status of participant recruitment, and observed adverse events.

### Adverse event reporting and harms {22}

Psychotherapy is usually not associated with risks, although reflecting personal problems can cause unpleasant or intense feelings. Based on the pilot study, researchers do not expect the study participants to be exposed to any risks or impairment due to our treatment or study procedures. Participants will receive state-of-the-art outpatient psychotherapy and complete established and standardized questionnaires. Both the control group (CBT) and the intervention group (EXP) will receive effective treatments.

There will be no special physical demands on study participants (no blood or saliva sampling, no medication or placebo administration, no invasive measurements). In the context of exposure treatment, a short-term increase in pain or muscle soreness may occur due to physical exercises (e.g., if participants are encouraged to become physically active again after a longer period of inactivity). In addition, symptoms of fatigue can occur due to completing the questionnaires.

Potential side effects of the treatment will be assessed via standardized self-report following every third therapy session. In addition, therapists will complete a checklist after every third therapy session to record adverse events and/or events that may influence the course of the treatment. The following events will be assessed systematically: (1) worsening of physical or mental symptoms, (2) admission to a somatic or psychiatric hospital, (3) starting an inpatient rehabilitation therapy, (4) interruption of therapy for more than 4 weeks, and (5) particularly stressful events in the participants’ private and/or professional environment.

In consultation with the supervisor, the therapist will evaluate the severity of occurring adverse events (mild, moderate, severe) and initiate appropriate steps, such as treatment interruption or medical consultation. In addition, the study’s medical supervisor, Prof. Dr. Ulrike Bingel from the Department of Neurology at the University Hospital Essen, will be available to be consulted for the clarification of red flags or dealing with medical adverse events.

### Frequency and plans for auditing trial conduct {23}

The DSMB will meet every 6 months to ensure that any imbalances between the intervention groups, e.g., dropouts, side effects, or protocol deviations, will be detected early. Clinical monitoring will follow a study-specific monitoring manual.

On every site, pre-study visits will be conducted by the clinical monitoring. All subsequent visits will depend on regularly scheduled feedback received by the monitor from the central monitoring, performed by the IMBI, and from the centers and study coordinators.

### Plans for communicating important protocol amendments to relevant parties (e.g., trial participants, ethical committees) {25}

All relevant changes that may affect the implementation of the study, study material, or participant safety will be reported to the ethical committees of the leading center and to the ethical committees of the recruitment centers in the form of a formal amendment. Any amendments or changes will be communicated to all parties involved in the conduct of the study, including the study registry. In the case of changes that will directly affect participants, the patient information leaflet will be adapted, and all study participants will be informed.

### Dissemination plans {31a}

Researchers will communicate the study’s findings to the International Association for the Study of Pain (IASP) and its German and European sections, as well as to psychological organizations such as the German Psychological Society. The results will be reported according to the CONSORT statement and will be presented at national and international conferences to medical and psychological experts to improve awareness of effective pain treatments. In addition, the results will be published as part of the final report to the German Research Foundation and will be accessible to all those interested on their pages. Results will also be available on the ClinicalTrials.gov website. A short summary of the results and information on where these can be accessed will be made available to interested participants. Also, these findings will be of interest to health insurance companies and the German pension insurance. The treatment manuals, if proven effective, will be offered to the public (open access). Finally, further data may be shared upon reasonable request and after all publication processes of our results will be completed.

## Discussion

To our knowledge, this study will be the first RCT to compare CBT and EXP treatment in chronic back pain, including patients from different healthcare structures and contexts. The results of the pilot study suggest that both treatments will improve pain-related distress.

Various measures, such as specific training in the application of treatment manuals, ongoing supervision of study therapists, and the participation of the patient’s primary care physicians, for example, in the clarification of red flags, ensure that our participants will receive high-quality therapy within a manageable time effort.

This study addresses the question of whether EXP is more effective than CBT; in addition, this research aims to investigate potential treatment-specific predictors of symptom improvement. Thus, it is expected that our trial will provide important starting points for future studies investigating customized treatments for chronic pain.

Furthermore, the study will lead to a larger number of therapists trained in EXP, thus strengthening outpatient treatment services for patients with CBP and increasing the visibility of a time- and cost-efficient treatment approach.

In addition, the involvement of both, psychological and medical centers, will promote interdisciplinarity, benefiting practitioners in the form of multi-professional exchange and patients in the form of overlapping and interlinked treatments. At the same time, the diversity of the centers will reflect the reality of the German health system with different settings and regional implications.

The structural diversity of the different centers (medical versus psychological) may be a limitation, as some processes and general conditions cannot be standardized in detail such as therapeutic rooms and allocation to therapists. However, center-specific differences will be considered in the analyses, and it is expected that the advantages for clinical practice will outweigh the disadvantages.

In the short term, an efficient and effective treatment rationale (EXP) should gain attention and become more widespread. In the long term, the plan is to offer customized treatment options for patients with chronic back pain and, thus, improve health care of CBP and individual disease-related impairments.

## Trial status

The recruitment phase started in June 2022 and is planned until the beginning of 2024. An application is currently being prepared with the sponsor to extend the duration of the study so that, if approved, recruitment should be completed in January 2025. As the study protocol was amended to include two additional questionnaires, the current protocol version is 2.0, which was approved on July 22, 2022. The end of the study is planned for the beginning of 2025. If the 1-year extension is approved, the study will end at the beginning of 2026.

## Data Availability

Until the publication of the study results, only the principal investigator and participating institutions such as IMBI have access to the cleaned data set. Non-identifiable data sets can be made available by the principal investigator upon reasonable request and after publication of study results. There are no contractual agreements.

## Abbreviations

CBP: Chronic back pain
CBT: Cognitive behavioral therapy
EXP: Graduated exposure in vivo
RCT: Randomized controlled trial
TAU: Treatment as usual
DSF: German Pain Questionnaire (Deutscher Schmerzfragebogen)
GEEE: Generic rating scale for previous treatment experiences, treatment expectations, and treatment effects
QBPDS: Quebec Back Pain Disability Scale
Mini DIPS: Diagnostisches Kurzinterview bei psychischen Störungen
LPFS-BF: Level of Personality Functioning Scale - Brief Form
PID5BF+M: Personality Inventory for DSM-5 - Brief Form Plus
PDI: Pain Disability Index
FESV: Fragebogen zur Erfassung der Schmerzverarbeitung
HADS: Hamilton Anxiety and Depression Scale
PCS: Pain Catastrophizing Scale
PHODA-SeV: Photograph Series of Daily Activities - Short Electronic Version
PASS-20: Pain Anxiety Symptom Scale
BAT-BACK: Behavioral Avoidance Test—Back Pain
PIPS: Psychological Inflexibility in Pain Scale
PFB-K: Partnerschaftfragebogen Kurzform
WAI-SR: Working Alliance Inventar short revised
REV: Rating of Expectancy Violation
INEP: Inventar zur Erfassung negativer Effekte von Psychotherapie
IMBI: Institute of Medical Biometry
eCRF: Electronic case report form
REDCap: Research Electronic Data Capture
SOP: Standard operating procedure
PMM: Predictive mean matching
PI: Principal investigator
DSMB: Data Safety Monitoring Board
IASP: International Association for the Study of Pain
JT: Jacobson-Truax method
